# The Rising Tide of Stimulant-Related Morbidity and Mortality Warrants Evidence-Based Treatment

**DOI:** 10.1177/07067437221125301

**Published:** 2022-09-15

**Authors:** Anees Bahji, Arash Dhaliwal, Arushi Sachdev, Marlon Danilewitz, Wiplove Lamba, Tony P. George, Nitin Chopra, David Crockford

**Affiliations:** 1 Department of Psychiatry, 2129 University of Calgary, Calgary, AB, Canada; 2 Department of Community Health Sciences, 2129 University of Calgary, Calgary, AB, Canada; 3 Hotchkiss Brain Institute, 2129 University of Calgary, Calgary, AB, Canada; 4 558158 British Columbia Centre on Substance Use, Vancouver, BC, Canada; 5 Department of Psychiatry, Schulich School of Medicine & Dentistry, 6221 Western University, London, ON, Canada; 6 School of Medicine, Queen’s University, Kingston, ON, Canada; 7 Department of Psychiatry, 7938 University of Toronto, Toronto, ON, Canada; 8 25487 Ontario Shores Center for Mental Health Sciences, Whitby, ON, Canada; 9 Department of Psychiatry, Temerty Faculty of Medicine, University of Toronto; Addictions Division, Centre for Addiction and Mental Health (CAMH), Toronto, ON, Canada

**Keywords:** addictions, substance use disorders, stimulants

The perspective of Bisaga *et al*. on treatment for individuals with psychostimulant use disorder (PSUD) is controversial.^1^ The authors propose an approach to treating PSUD akin to opioid use disorder (OUD), arguing that stimulant agonist medications could transform existing drug treatment paradigms. The authors conclude that the evidence supports prescription stimulant agonists in combination with psychosocial interventions. While the authors discussed potential safety concerns and adverse effects, such as cardiovascular, neurological, psychotic and mood symptoms, we disagree with the authors’ interpretation of these findings.

Although there have been many reviews of PSUD pharmacotherapies, the only one to arrive at a positive conclusion was the meta-analysis by Tardelli *et al*. 2020,^2^ and consequently, the only review cited by Bisaga *et al*. to support their claim. Yet several meta-analyses have mostly noted the lack of consistent evidence for any pharmacological intervention for PSUD, emphasizing the low-grade quality of evidence, high dropout rates, no improvements in treatment retention, and possible adverse reactions and side effects from exposing vulnerable individuals to high-dose psychostimulants.^3^ Moreover, a more recent network meta-analysis comparing all pharmacological and psychological treatments for cocaine use disorder found that contingency management was the only treatment with significant efficacy at increasing the likelihood of having a negative test result for the presence of cocaine.^4^ This finding is especially salient as it highlights a distinction between paradigms for treating OUD where replacement therapy is paramount over therapy.

Unlike OUD, the available evidence for agonist-based pharmacological treatment of PSUD has been inconsistent, and there is no evidence of reduced mortality.^4^ Although some trials have suggested that psychostimulants might be an effective strategy for PSUD treatment, multiple Cochrane Collaboration reviews on PSUD treatment concluded that since treatment dropout was high in many RCTs, the appearance of high stimulant abstinence (i.e., negative urine drug screens) could be explained by attrition bias instead.^3^

We appreciate the need for advocacy given the limited treatment offerings for patients with PSUD. Still, Bisaga *et al*. did not emphasize the lack of consistent benefit shown in RCTs and meta-analyses of prescription psychostimulants and other pharmacotherapies for PSUD,^3^ instead choosing to view the literature around psychostimulants as unequivocally positive. In addition, the risk of precipitating or worsening psychosis with the prescription of stimulants is minimally addressed in their argument, even though psychosis occurs in 36.5% of persons with methamphetamine use disorder.^5^ They also suggest that prescription stimulants could lower the risk of relapse, despite there being robust neurobiological evidence identifying that priming with either the primary drug, drugs of a similar class, or drugs that activate shared pathways could reinstate drug craving, seeking and use.^5^

We agree with Bisaga *et al*. that the problems with PSUD are urgent, and there must be well-conducted treatment trials for persons with PSUD. However, the evidence for a prescription agonist approach to date is lacking and poses potential risks for inadvertently worsening PSUD treatment outcomes by precipitating/worsening psychosis and increasing relapse to psychostimulant use. Furthermore, emphasizing stimulant replacement may further misdirect clinicians from pursuing treatment options with more robust evidence, namely contingency management. Therefore, communities that seek solutions should wait for confirmatory efficacy studies before implementing the approach proposed by Bisaga *et al*. for persons with PSUD, exercising caution and increasing access to evidence-based psychosocial interventions with appropriate wrap-around services.
